# 

**Published:** 1933

**Authors:** 


### CONTENTS.

| | Page |
|---|---|
| **A Surgical Adventure: An Autobiographical Sketch.** By Ernest W. Hey Groves, M.S., F.R.C.S. | 1 |
| **Enlarged Prostate.** By A. Rendle Short, M.D., B.S., F.R.C.S. | 23 |
| **Anterior Poliomyelitis, or Infantile Paralysis.** By H. Chitty, M.S., F.R.C.S. | 37 |
| **Reviews of Books** | 51 |
| **Editorial Notes** | 57 |
| **Obituaries.** William Henry Harsant, F.R.C.S. | 60 |
| Christopher Elliott, M.D. | 62 |
| Sidney John Hermann Griffiths, M.B., Ch.B., L.R.C.P., F.R.C.S. | 62 |
| Mrs. Evelyn Rachel Bates, M.R.C.S., L.R.C.P. | 63 |
| **Meetings of Societies** | 65 |
| **The Medical Library of the University of Bristol** | 66 |
| **Publications Received** | 70 |
| **Local Medical Notes** | 71 |

